# Di-2-pyridylhydrazone Dithiocarbamate Butyric Acid Ester Exerted Its Proliferative Inhibition against Gastric Cell via ROS-Mediated Apoptosis and Autophagy

**DOI:** 10.1155/2018/4950705

**Published:** 2018-03-25

**Authors:** Xingshuang Guo, Yun Fu, Zhuo Wang, Tingting Wang, Cuiping Li, Tengfei Huang, Fulian Gao, Changzheng Li

**Affiliations:** ^1^Department of Histology and Embryology, Xinxiang Medical University, Xinxiang, Henan 453003, China; ^2^Laboratory of Molecular Medicine, Xinxiang Medical University, Xinxiang, Henan 453003, China; ^3^Department of Molecular Biology and Biochemistry, Xinxiang Medical University, Xinxiang, Henan 453003, China

## Abstract

Diversified biological activities of dithiocarbamates have attracted widespread attention; improving their feature or exploring their potent action of mechanism is a hot topic in medicinal research. Herein, we presented a study on synthesis and investigation of a novel dithiocarbamate, DpdtbA (di-2-pyridylhydrazone dithiocarbamate butyric acid ester), on antitumor activity. The growth inhibition assay revealed that DpdtbA had important antitumor activity for gastric cancer (GC) cell lines (IC_50_ = 4.2 ± 0.52 *μ*M for SGC-7901, 3.80 ± 0.40 *μ*M for MGC-803). The next study indicated that growth inhibition is involved in ROS generation in mechanism; accordingly, the changes in mitochondrial membrane permeability, apoptotic genes, cytochrome *c*, bax, and bcl-2 were observed, implying that the growth inhibition of DpdtbA is involved in ROS-mediated apoptosis. On the other hand, the upregulated p53 upon DpdtbA treatment implied that p53 could also mediate the apoptosis. Yet the excess generation of ROS induced by DpdtbA led to cathepsin D translocation and increase of autophagic vacuoles and LC3-II, demonstrating that autophagy was also a contributor to growth inhibition. Further investigation showed that DpdtbA could induce cell cycle arrest at the G1 phase. This clearly indicated the growth inhibition of DpdtbA was via triggering ROS formation and evoking p53 response, consequently leading to alteration in gene expressions that are related to cell survival.

## 1. Introduction

Gastric cancer (GC) ranks as one of the fifth most common malignancies in the world, and more than half of cases are reported annually in East Asia [1, 2]. Resection may benefit certain patients, but mostly transiently due to metastasis. Chemotherapy is still the main treatment for advanced GC [3]. It has been demonstrated that 5-year overall survival increased by 6% with chemotherapy compared to that of surgical treatment alone [4]. However, the side effects and resistance of chemotherapeutic agents limit their wide use, thus requiring alternative drugs.

Enzymes are important biological macromolecules involved in many biological processes. Almost half of all enzymes associate with a particular metal ion to function [5], such as cytochrome oxidase, zinc-copper superoxide dismutase, and lysyl oxidase, and several transcription factors require copper for activity [6]. It is well documented that cancer cells have an increased demand for iron and copper to maintain robust cell proliferation and metastasis; thus, disturbing the metal requirement of cancer cells by chelators has been an alternative option for cancer therapy [7].

Dithiocarbamates have received attention because of its multiple biological activities and strong affinity toward metal ions, which aroused an interest to probe their potent applications on disease treatment [8]. However, the strong affinity toward transition metals, especially to copper and zinc ions, may also bring undesirable consequences, such as direct inactivation of enzymes that are required for cell growth [9–11]. To achieve the optimal therapeutic index, a reasonable balance is needed between cytotoxicity and affinity to metal ions for dithiocarbamates; thus, structural modification of dithiocarbamate is an active field. Dithiocarbazate is a widely used intermediate that can react with aldehyde and ketone derivatives, as well as alkylating agents; the resulting dithiocarbamate derivatives are more stable. We previously reported that *S*-propionic or acetic acid ester of di-pyridylhydrazone dithiocarbamate had excellent antitumor activity but has lesser activity for their copper complexes; this situation was rare in literatures [12, 13]. Analysis of structure-activity relationship revealed that *S*-propionic acid of the dithiocarbamate was better than *S*-acetic acid; however, a reversed phenomenon was found for their complexes in growth inhibition, which aroused an interest to further probe the effect of length of carbon chain on biological activity. To extend our knowledge of the new dithiocarbamate derivatives, in this study, di-pyridylhydrazone dithiocarbamate *S*-butyric acid (DpdtbA) was further prepared and characterized by MS and NMR. Next, its antiproliferative effect was evaluated on gastric cancer cell lines, as like other dithiocarbamates we have reported previously, the new prepared DpdtbA also showed excellent antitumor activity; thus, its underlying mechanism was preliminarily investigated. In vitro ROS assay revealed that DpdtbA could induce ROS generation, which triggered p53 response; consequently, the apoptotic genes and externalization of phosphatidylserine were altered, supporting the finding that induced apoptosis was p53 mediated. The excess ROS generated by DpdtbA also caused autophagy, protease leakage from lysosome, and cell cycle arrest, implying that ROS played important roles in the proliferation inhibition.

## 2. Results

### 2.1. Proliferation Inhibition of DpdtbA

**Figure 1:**
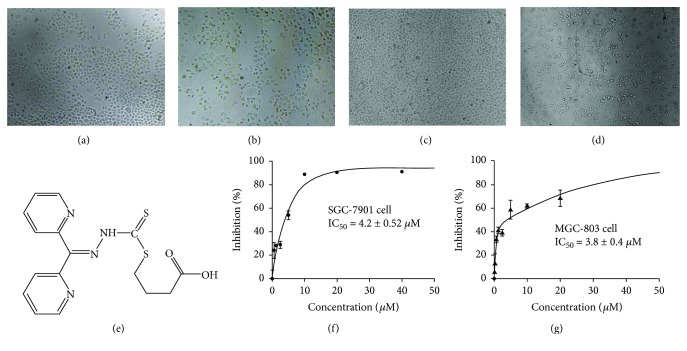
The chemical structure and growth inhibition of DpdtbA. The effect of DpdtbA on morphology. SGC-7901 cells: (a) control and (b) 5 *μ*M DpdtbA after 16 h treatment. MGC-803 cells: (c) control and (d) 5 *μ*M DpdtbA treatment after 16 h treatment. (e) Structure of DpdtbA. (f) Proliferation inhibition of DpdtbA against SGC-7901 cell line (IC_50_ = 4.2 ± 0.52 *μ*M). (g) Proliferation inhibition of DpdtbA against MGC-803 cell line (IC_50_ = 3.8 ± 0.4 *μ*M).

DpdtbA was prepared as described previously [12], with the difference being that 4-bromine butyric acid was used in the last step-reaction. The resulting dithiocarbamate, di-2-pyridylhydrazone dithiocarbamate butyric acid ester (DpdtbA), was characterized by NMR and MS spectra (see Materials and Methods); the structure of DpdtbA is shown in Figure 1(e). HPLC and NMR indicated that it has adequate purity (>95%) for biological assay (Figure S1). Next, we assessed the growth inhibition of DpdtbA against gastric cancer cell lines, SGC-7901 and MGC-803; the dose-response curves are depicted in Figure 1. As shown in Figures 1(f) and 1(g), DpdtbA had significant growth inhibition with IC_50_ = 4.20 ± 0.52 *μ*M for SGC-7901 and 3.80 ± 0.40 *μ*M for MGC-803. The cell dependence was not obvious, but the maximal inhibition was slightly different. For SGC-7901 cells, the maximal inhibition (~80%) was achieved at ~10 *μ*M, but 40 *μ*M DpdtbA is required for same inhibition of MGC-803 (Figures 1(f) and 1(g)). The morphology changes when exposure of the agent to the investigated cells was also recorded; rounded cells were observed for both cell lines (compare Figures 1(b) and 1(d) with Figures 1(a) and 1(c)).

### 2.2. DpdtbA Induced a ROS-Dependent Growth Inhibition

**Figure 2:**
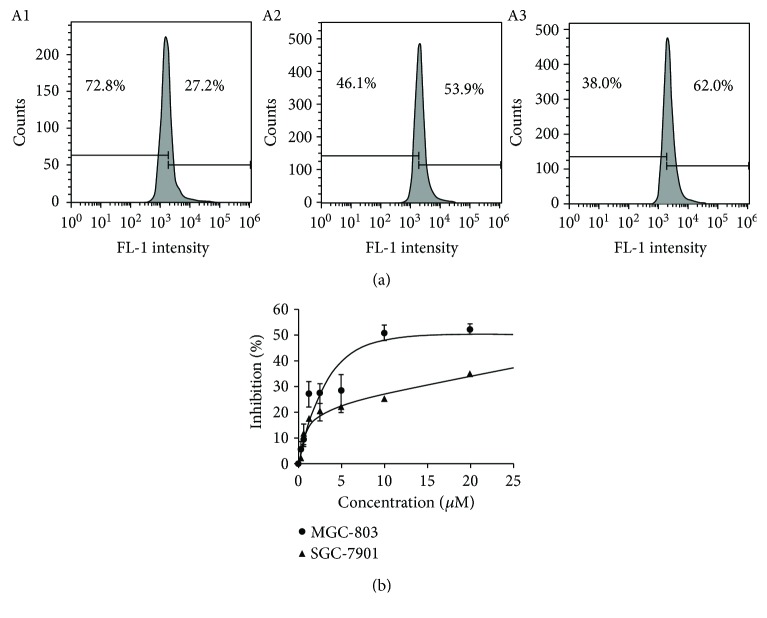
DpdtbA induced ROS generation and growth inhibition in the presence of NAC. (a) MGC-803 cells used for ROS assay (24 h incubation): (A1) DMSO, (A2) 2.5 *μ*M DpdtbA, and (A3) 5 *μ*M DpdtbA. (b) Growth inhibition of DpdtbA in the presence of 1.5 mM NAC.

Generation of reactive oxygen species (ROS) in mechanism is involved in many chemotherapeutic agents; thus, the ability of ROS production was assessed. DCFH-DA is frequently used to determine ROS; the populations in different fluorescence intensities were measured by flow cytometry. As shown in Figure 2, ROS increased with increasing DpdtbA (Figure 2(A2 and A3)). To determine whether ROS correlated with growth inhibition, a ROS scavenger, NAC (*N*-acetyl-L-cysteine), was introduced into the growth inhibition assay. As shown in Figure 2(b), the inhibitory effect of DpdtbA against both cell lines in the presence of NAC was significantly attenuated. For SGC-7901 cell, DpdtbA caused ~85% growth inhibition at low concentration (10 *μ*M) but ~30% growth inhibition at higher concentration (20 *μ*M) in the presence of 1.5 mM NAC (Figures 1(f) and 2(b)); similar for MGC-803 cell, IC_50_ was increased by ~3-fold in the presence of NAC (Figures 1(g) and 2(b)). This clearly indicated that the growth inhibition displayed by DpdtbA was ROS dependent.

### 2.3. DpdtbA and Its Copper Complex Induced Cellular Apoptosis

**Figure 3:**
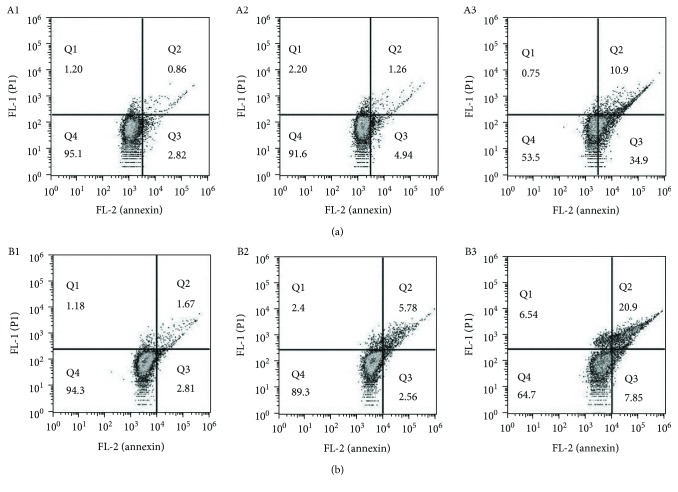
Flow cytometry analysis of apoptosis of GC cell lines. DpdtbA was incubated with the cells for 24 h. All attached cells were collected and double stained with annexin V and propidium iodide (PI) using a kit from Dojindo Laboratories following the manufacturer's instructions. (a) SGC-7901: (A1) DMSO, (A2) 2.5 *μ*M DpdtbA, and (A3) 5.0 *μ*M DpdtbA. (b) MGC-803: (B1) DMSO, (B2) 2.5 *μ*M DpdtbA, and (B3) 5.0 *μ*M DpdtbA.

The excess intracellular ROS correlating with apoptosis has been well documented; the elevated ROS level implies that the action of the agent may be involved in apoptosis induction. To measure the apoptosis populations at early and late stages, the annexin V/propidium iodide (PI) staining was performed, which measures externalization of phosphatidylserine on the cell surface of apoptotic cells specifically. As shown in Figure 3, DpdtbA induced early apoptosis and later apoptosis of GC cells in a concentration-dependent manner; that is, the population of apoptosis was increased from 4.8 to 46.5% for SGC-7901 cells and from 5.6 to 35.0% for MGC-803 cells (Figures 3(a) and 3(b)).

**Figure 4:**
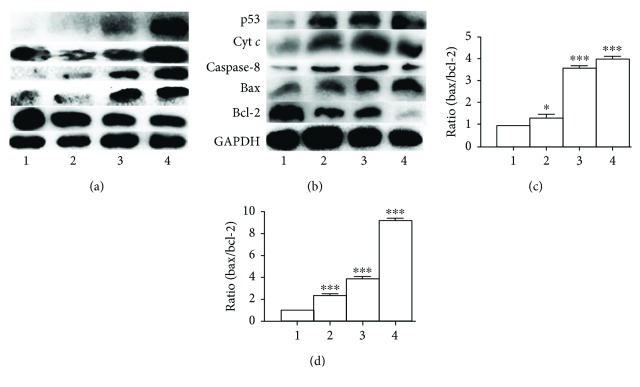
Western blotting analysis of changes of apoptosis-related genes. (a) MGC-803 and (b) SGC-7901: 1 = DMSO; 2 = 1.25 *μ*M DpdtbA; 3 = 2.5 *μ*M DpdtbA; 4 = 5 *μ*M DpdtbA. (c) Normalized ratio of bax/bcl-2 (MGC-803). (d) Normalized ratio of bax/bcl-2 (SGC-7901) (^∗^ *p* < 0.05; ^∗∗∗^ *p* < 0.01; one-way ANOVA).

To further support involvement of apoptosis, Western blotting was employed to determine changes in apoptotic genes. The excess ROS generally lead to apoptosis through changes in the expression of bcl-2 family proteins; thus, the alterations of bcl-2 and bax proteins before and after treatment with DpdtbA at different concentrations were measured. As shown in Figure 4, the upregulated bax and downregulation Bcl-2 were observed when DpdtbA treated SGC-7901 and MGC-803 cells in contrast to that of control (Figures 4(a) and 4(b)). Accordingly, the relative ratios of bax/bcl-2 were also generated for comparative purposes; clearly, the related ratios were elevated with increased concentration of DpdtbA (Figures 4(c) and 4(d)). In addition, the other apoptotic genes, caspase-8 and cytochrome *c*, were also observed to be upregulated; this was in agreement with the result of externalization of phosphatidylserine from flow cytometry (Figure 3), implying that the apoptosis was involved in the growth inhibition caused by DpdtbA.

**Figure 5:**
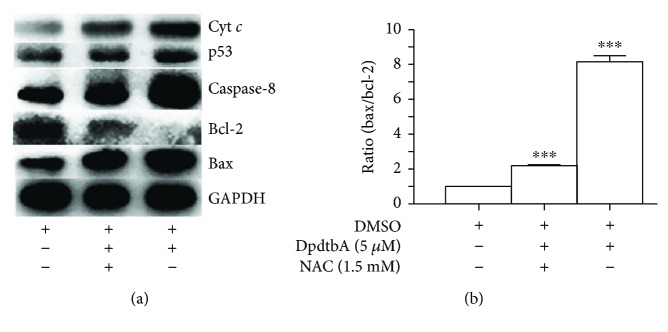
The effect of NAC on expressions of apoptotic genes of MGC-803 cells. (a) Western blotting analyses of apoptotic genes; (b) the changes in ratio of bax/bcl-2 in the presence or absence of NAC (^∗∗∗^ *p* < 0.01; one-way ANOVA).

To determine whether the alterations of apoptotic proteins correlated with ROS generation, the decreased ratio of bax/bcl-2 would be indicative when scavenging ROS. Thus, a ROS scavenger, NAC (*N*-acetyl-L-cysteine), was employed. In view of a similar tendency toward DpdtbA treatment in both gastric cell lines, the MGC-803 cell line was chosen and treated by either DpdtbA alone or combined with NAC; the related changes of the apoptotic genes are shown in Figure 5. It was clear that the NAC did decrease the ratio of bax/bcl-2 in contrast to the DpdtbA treatment only (Figure 5(b)). In addition, the other apoptosis-related proteins, cyt *c* and caspase-8, were also downregulated upon addition of NAC. Similarly, DpdtbA changed the mitochondrial membrane permeability (Figure S2); this indicated that the agent-induced apoptosis was ROS dependent. Since ROS is involved in growth inhibition, in response to ROS, p53 might be activated; upregulation of p53 when treated by the agent was observed but downregulation in combination with NAC (Figure 5(a)), implying that p53 played a role in the growth inhibition.

**Figure 6:**
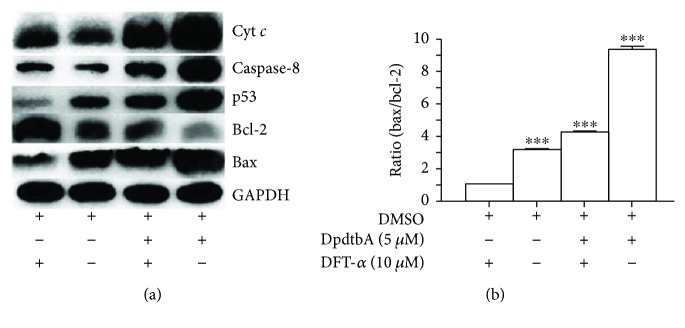
p53 played a role in DpdtbA-induced apoptosis. (a) Western blotting analyses of apoptotic genes when the MGC-803 cells treated with either DpdtbA alone or combined with p53 inhibitor; (b) the changes in ratio of bax/bcl-2 in different conditions (^∗∗∗^ *p* < 0.01; one-way ANOVA).

To further determine the role of p53 in the growth inhibition, a p53 inhibitor, pifithrin-*α* (PFT-*α*), was employed; accordingly, the MGC-803 cells were treated with either DpdtbA alone or in combination with PFT-*α*; p53 and other apoptotic proteins were evaluated by immunoblotting. As shown in Figure 6, the addition of PFT-*α* attenuated the increases of p53, cyt *c*, caspase-8 (Figure 6(a)), and ratio of bax/bcl-2 (Figure 6(b)), indicating that p53 did play a role in the process of induced apoptosis.

### 2.4. DpdtbA Induced Change in Lysosomal Membrane Permeability (LMP) and Autophagy Response

**Figure 7:**
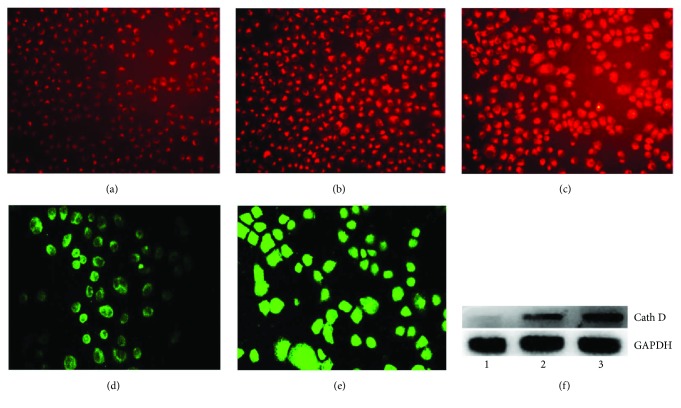
DpdtbA-induced change in lysosomal membrane permeability and cathepsin D translocation. LysoTracker Red-stained MGC-803 cells (objective size 10 × 10): (a) control, (b) 2.5 *μ*M DpdtbA, and (c) 5.0 *μ*M DpdtbA. The enhanced fluorescence intensities of the cells clearly indicated the alteration of LMP. Immunofluorescence detection of cathepsin D in MGC-803 cells (objective size 10 × 20): (d) control cells and (e) 2.5 *μ*M DpdtbA. (f) Western blotting analysis of cathepsin D in cytosol.

Lysosome is a subcellular compartment that contained a host of hydrolytic enzymes and is responsible for digesting long-lived proteins and organelles, so the lysosomal membrane integrity is an important factor for maintaining its function. Some chemotherapeutic agents caused apoptosis and alteration of lysosomal membrane permeability; DpdtbA might have a similar action. To test the hypothesis, LysoTracker Red that can accumulate within lysosomes was employed to assess the lysosome membrane permeability [13]. As shown in Figure 7, the red fluorescence intensities of MGC-803 cells increased when DpdtbA was increased compared to those of nontreated cells, indicating that more LysoTracker Red accumulated in lysosomes and LMP was altered (Figures 7(b) and 7(c)). Since the alteration of the permeability, we questioned whether cathepsin release also occurred. To determine the possibility, cathepsin D was evaluated by immunofluorescence technique. As shown in Figure 7, the granular-stained cathepsin D was observed in the untreated cells (Figure 7(d)) and a diffusion pattern in the DpdtbA-treated cells (Figure 7(e)), indicating that cathepsin D was released from lysosome to cytosol, implying that apoptosis has occurred. A similar result from a Western blotting analysis further supported the increase of cathepsin D in cytosol, consistent with that reported previously [14].

**Figure 8:**
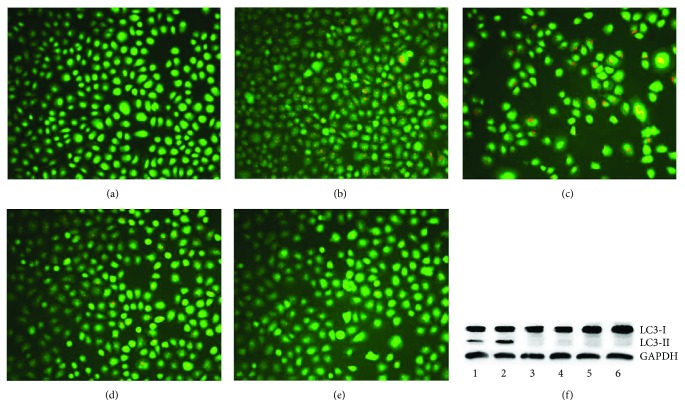
DpdtbA-induced autophagy in MGC-803 cells. Stained by acridine orange: (a) control, (b) 2.5 *μ*M DpdtbA, (c) 5 *μ*M DpdtbA, (d) 2.5 *μ*M DpdtbA + 3-MA (2 mM), and (e) 5 *μ*M DpdtbA + 3-MA (2 mM). (f) Western blotting: 1 = 5 *μ*M DpdtbA plus 1.5 *μ*M NAC; 2 = 5 *μ*M DpdtbA; 3 = 5 *μ*M DpdtbA plus 1.5 mM 3-MA; 4 = DMSO control; 5 = 1.5 mM NAC; 6 = 1.5 mM 3-MA.

Apoptosis associated with release and translocation of cathepsin has been realized [15], which may have stemmed from induced ROS generation. DpdtbA could produce excess ROS in response to the oxidative stress, and a response to autophagy might occur. Thus, the formation of autophagosome was measured by acridine orange staining. As shown in Figures 8(a)–8(c), the red granular fluorescence in the acidic vacuoles was observed in the agent-treated groups and in a concentration-dependent manner, indicating that more autophagic vacuoles were formed. To confirm the above result, 3-methyladenine (3-MA), an autophagy inhibitor, was introduced in the assay; clearly, the red granular fluorescence in the acidic vacuoles decreased in contrast to that of the agent only (Figures 8(b)–8(e)). To ensure the reliability of the results, the formation of autophagic vacuoles or autophagosomes was further analyzed by monodansylcadaverine (MDC) staining via flow cytometer and microcopy techniques (Figures S3 and S4) [16]. As shown in Figure S3, the fluorescence intensities of MDC were increased with treatment of DpdtbA and decreased with addition of 3-MA or NAC, and the trend in fluorescence intensity was similar to that of acridine orange staining (Figures 8(a)–8(e)). A similar situation was found during morphologic observation (Figure S4). This supported that autophagy occurred. Furthermore, the molecular evidence of autophagy occurrence was from measurement of LC3-II (microtubule-associated protein light chain 3), an autophagosome marker. As expected, the increase of LC3-II was observed when DpdtbA was exposed to the cells (Figure 8(f), line 2), but with addition of 3-MA or NAC, the LC3-II decreased or disappeared (Figure 8(f), line 1, line 3), indicating that DpdtbA indeed induced autophagy (Figure 8).

### 2.5. The Effect of DpdtbA on Cell Cycle

**Figure 9:**
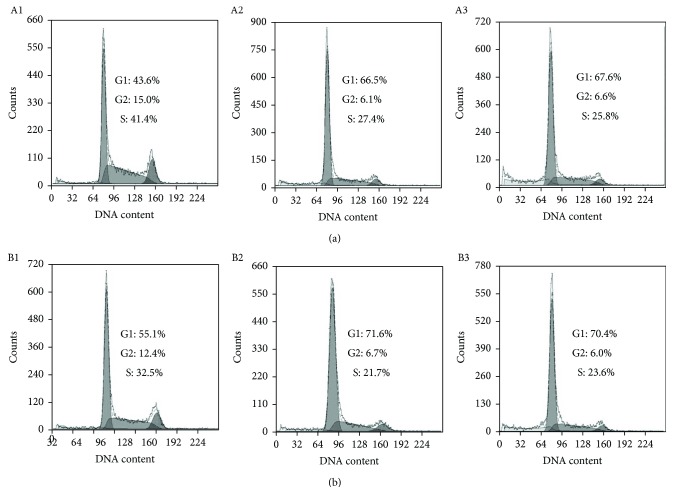
Cell cycle distribution of GC cells following treatment with various concentrations of DpdtbA. (a) SGC-7901: (A1) control, (A2) 2.50 *μ*M DpdtbA, and (A3) 5.0 *μ*M DpdtbA. (b) MGC-803: (B1) control, (B2) 2.5 *μ*M DpdtbA, and (B3) 5.0 *μ*M DpdtbA.

ROS induce cell cycle delay at the G1/S boundary [17]. We therefore evaluated the effect of DpdtbA on the cell cycle distribution using propidium iodide staining via flow cytometry. As shown in Figure 9, DpdtbA caused an accumulation of the GC cells in the G1 phase, and the percentage at the G1 phase significantly increased from 43.6 to ~70% during 24 h insult of the agent with both cell lines (Figures 9(a) and 9(b)), indicating that DpdtbA can disturb cell cycle and arrest the cells at the G1 phase as do other iron chelators [18].

## 3. Discussion

Transition metals, such as iron, that require to maintain viability and to support proliferation of almost all kinds of cells [19] play important roles in biosystem. Those metals locate either in proteins as cofactor or in cytosol as free form in metal labile pool. Clearly, chelators can disturb homeostasis of the metals, accordingly producing different biological effects. Cancer cells have higher iron demand than have normal cells, and depleting iron will be favorable to inhibit proliferation of cancer cells; thus, chelation therapy is a promising strategy. Dithiocarbamate derivatives have strong affinity toward transition metals; pyrrolidine dithiocarbamate (PDTC) is a representative compound, exhibiting diverse biological effects [20–22]. In view of instability and stronger affinity toward transition metals, especially zinc and copper ions, the thiol-modified dithiocarbamate derivatives were prepared to enhance their stability and improve their biological activity [23, 24]. In the present study, a new synthesized dithiocarbamate derivative (DpdtbA) showed better antitumor activity against gastric cell lines. In view of its good activity, the underlying mechanism was preliminarily investigated. Generally, antitumor drugs used clinically exert one of their actions via generating excess ROS, which causes oxidative damage of protein and nucleic acids, consequently resulting in cell death [25, 26]. DpdtbA may have a similar action. As expected, DpdtbA indeed has the ability to generate ROS at a cellular level (Figure 2). Next, we questioned whether the increased ROS correlated to the growth inhibition; thus a ROS scavenger, NAC, was employed in the proliferation assay. Clearly, addition of NAC could attenuate the cytotoxicity of DpdtbA (or increase viability of gastric cells), indicating that there was a ROS-dependent growth inhibition, which was in agreement with that reported previously [27, 28]. The correlation between ROS and apoptosis has been well investigated; one of the molecular events upon apoptosis is externalization of phosphatidylserine, which can be revealed by the annexin V/propidium iodide (PI) staining though flow cytometric analysis [29]. The cytometric data showed that the DpdtbA induced early apoptosis and later apoptosis in a concentration-dependent manner (Figure 3). The evidence from Western blotting analysis also supported involvement of apoptosis due to the upregulation of bax, p53, and caspases and downregulation of bcl-2 after DpdtbA exposure to the cells (Figure 4). This situation is frequently observed in cells subjected to drug treatments [30]. The aforementioned changes in the apoptotic proteins may stem from ROS generation. To conform to the relevance, an antioxidant, NAC, was used to scavenge ROS; accordingly the extent of apoptosis was attenuated, indicating that ROS indeed play an important role in apoptosis induced by DpdtbA (Figure 5). p53 is a housekeeper gene that responds to external stimulus or oxidative stress; the upregulated p53 implied that it might be involved in the regulation of expression of bcl-2 family [31]. In this study, DpdtbA upregulated p53 protein expression, downregulated antiapoptotic protein bcl-2 expression, promoted proapoptotic protein bax expression, and triggered cell apoptosis via intrinsic and extrinsic pathways (Figure 6) [32]. Moreover, p53 can directly induce bax and bak oligomerization [33]; thus, the higher ratio of bax/bcl-2 would be favorable for bax oligomerization, consequently translocating the oligomerized bax to the mitochondrial membrane, which releases cytochrome *c* and causes mitochondrial cell death. Some forms of apoptosis have been found to be associated with a lysosomal pathway [34]. Similarly, the translocation of bax oligomer to lysosomal membrane causing the release of cathepsins from the lysosomal lumen to the cytosol has been also observed [35, 36]. Cathepsin D normally resides within lysosomes and endosomes but can be translocated to the cytoplasm under stress conditions, where it initiates apoptosis [37]. In the present study, this phenomenon of translocation of cathepsin D was also found (Figure 7), which was consistent with that reported previously [38]. The relocation of lysosomal cathepsins induces apoptotic signaling and leads to lysosomal cell death [39]; this clearly indicated that growth inhibition induced by DpdtbA is involved in apoptosis.

Autophagy plays an important role in cell survival by removing misfolded or aggregated proteins, clearing damaged organelles, and eliminating intracellular pathogens [40]. ROS-triggered apoptosis and autophagy have been well documented [41]. DpdtbA induced excess ROS production; accordingly, autophagy may be also involved. Figures 8(b) and 8(c) showed that the accumulated red granular fluorescence in the acidic vacuoles in DpdtbA-treated cells was increased but decreased with the addition of an autophagy inhibitor (3-MA) (Figures 8(d) and 8(e)); a similar result was from flow cytometric analysis based on MDC staining (Figure S3). These data clearly showed that autophagosomes were increased; that is, autophagy was activated when DpdtbA was exposed to the cells. Interestingly, with the addition of a ROS scavenger, NAC, autophagy induced by DpdtbA was attenuated, indicating that occurrence of autophagy was triggered by ROS, which was consistent with findings of literatures [40, 41]. LC3 is an autophagosome molecular marker; the increase of LC3-II and decrease of LC3-I indicated that autophagy was activated. In the present study, DpdtbA induced the change in LC3, and the changes were attenuated by an addition of either autophagy inhibitor or ROS scavenger (Figure 8(f)). The aforementioned data demonstrated that autophagy made a contribution to the growth inhibition induced by DpdtbA. ROS generation leads to cell cycle delay. The cell cycle analysis revealed that DpdtbA could induce cell cycle arrest at the G1 phase as reported for other iron chelators (Figure 9) [18, 42]. Generally, iron chelators exhibit ribonucleotide reductase inhibition and then disturb cellular DNA synthesis [43]; therefore, cell cycle arrest caused by the agent was also partly a contributor to growth inhibition. It was noted that the DpdtbA-induced cell cycle arrest may be cell line dependent because its analogue, DpdtpA, led to cell cycle arrest in the S phase for hepatoma carcinoma cell line [12].

In conclusion, DpdtbA exhibited significant antiproliferative activity against gastric cells; it may stem from induction of ROS generation, subsequently provoking p53 response and triggering apoptosis, autophagy, lysosomal cell death, and cell cycle arrest. Therefore, ROS may be an initiator in the growth inhibition.

## 4. Materials and Methods

### 4.1. General Information

MTT, acridine orange, di-2-pyridylketone, pifithrin-*α*, and monodansylcadaverine (MDC) were purchased from Sigma-Aldrich. LC3 antibody was obtained from Proteintech Group (Wuhan, China), caspase-8, GAPDH, bax, cytochrome *c*, and bcl-2 were purchased from Boster (Wuhan, China). RPMI 1640 and fetal bovine serum were purchased from Zhejiang Tianhang Biological Technology Co. Ltd.

### 4.2. Preparation of Di-2-pyridylhydrazone Dithiocarbamate *S*-Butyric Acid (DpdtbA)

DpdtbA (chemical name generated by ACD/Labs, 3-[({2-[di(pyridin-2-yl)methylidene]hydrazinyl}carbonothioyl)sulfanyl]butyric acid) was made as reported previously [12]. Briefly, the hydrazine dithiocarbamate was synthesized by reaction of equimolar carbon disulfide (1 mmol) with hydrazine (1 mmol) in KOH containing ethanol (10 ml) on an ice bath for 1 h. Then the reaction mixture without further separation was mixed with equimolar di-2-pyridylketone (1 mmol); the resulting mixture was refluxed for 1 h. After being cooled, the red-brown solid was filtered and washed with cold ethanol. TLC showed one pot (ethyl acetate/petroleum ether = 3 : 1). Next, the red-brown (di-2-pyridylhydrazone dithiocarbamate, 1 mmol) was dissolved in absolute ethanol (5 ml) and reacted with 4-bromo butyric acid at room temperature for 1 h; the yellow solid was filtered and washed with ethanol. TLC traced (ethyl acetate/petroleum ether = 3 : 1). mp: 158.7°C. ^1^H NMR (Ascend™ 400 spectroscope, Bruker, Switzerland), ppm: 14.93 (s, NH), 8.86 (d, H, *J* = 4 Hz), 8.63 (d, H, *J* = 4 Hz), 8.03 (m, 2H, *J* = 8 Hz), 7.98 (d, H, *J* = 8 Hz), 7.64 (dd, H, *J* = 4 Hz), 7.60 (d, H, *J* = 8 Hz), 7.54 (dd, H, *J* = 4 Hz), 3.27 (tri, 2H, *J* = 8 Hz), 2.37 (tri, H, *J* = 8 Hz), 1.91 (tri, H, *J* = 8 Hz); ^13^C NMR (Ascend 400 spectroscope, Bruker, Switzerland), ppm: 199.98, 174.28, 155.08, 150.98, 149.18, 148.68, 138.34, 137.96, 128.10, 125.80, 124.93, 124.14, 33.10, 24.26. ESI-MS (C_16_H_16_N_4_S_2_O_2_ (cal/obs): M + Na: 383.0615 (383.0606).

### 4.3. Cytotoxicity Assay (MTT Assay)

A 10 mM DpdtbA in 80% DMSO was diluted to the required concentration with culture. The MTT assay was conducted as previously described [9]. Briefly, 5 × 10^3^/ml MGC-803 (or SGC-7901) cells in exponential phase was seeded equivalently into a 96-well plate, and the various amount of DpdtbA was added after the cells adhered. After 48 h incubation at 37°C in a humidified atmosphere of 5% CO_2_, 10 *μ*l MTT solution (5 mg/ml) was added to each well, followed by further 4 h incubation. The cell culture was removed, and 100 *μ*l DMSO was added in each well to dissolve the formazan crystals. The measurement of absorptions of the solution that was related to the number of live cells was performed on a microplate reader (MK3, Thermo Scientific) at 570 nm. Percent growth inhibition was defined as percent absorbance inhibition within appropriate absorbance in each cell line. The same assay was performed in triplicate. Morphologic study was conducted under inverted microscope (Shanghai Batuo Instrument Co. Ltd., Shanghai, China); the photographs of SGC-7901 and MGC-803 cells treated by DpdtbA (2.5 or 5.0 *μ*M for 16 or 48 h) were recorded (objective size: 10 × 20).

### 4.4. ROS Detection In Vivo

As described in MTT assay, the MGC-803 cells were treated by DpdtbA for 24 h. The cells were collected by centrifugation after trypsinization. Following PBS washing, the cell pellets were resuspended in serum-free culture medium containing H_2_DCF-DA and incubated for 30 min. Finally, the medium containing H_2_DCF-DA was removed by centrifugation and washed with PBS, and the cells were resuspended in PBS. The intracellular ROS assay was performed on a flow cytometer (Becton Dickinson, USA).

### 4.5. Flow Cytometric Analysis of Apoptosis

Cells were seeded into a 6-well plate and treated as described above for the cell viability assay. The cells were treated with different concentrations of the agent (2.5 and 5.0 *μ*M DpdtbA) for 24 h. Then the cell culture was removed, following PBS washing and trypsin digestion; finally, the annexin V and propidium iodide (a kit from Dojindo Laboratories, Kumamoto, Japan) were added as recommended by the company. The stained cells were subjected to cytoflow analysis (Becton Dickinson, USA).

### 4.6. Western Blotting Analysis

The protocol used for Western blotting was as previously reported [10]; briefly, 1 × 10^7^ MGC-803 (or SGC-7901) cells treated with or without DpdtbA was scraped off in lysis buffer (50 mM Tris-HCl, pH 8.0, 150 mM NaCl, 1.0% NP-40, 10% glycerol, and protease inhibitors) on ice for 30 min, following spin down by centrifugation at 14,000 ×g. The clear supernatant was stored at −80°C. The protein concentration was determined using a colorimetric Bio-Rad DC protein assay on a microplate reader MK3 at 570 nm. Proteins (30 *μ*g) were separated on a 13% sodium dodecyl sulfate-polyacrylamide gel at 200 V for 1 h. Then, the separated proteins were subsequently transferred onto a PVDF membrane at 60 V for 1 h. The membrane was washed three times with Tris-buffered saline (TBS) and was then blocked for 2 h in TBS containing 0.1% Tween-20 and 5% nonfat skimmed milk. The membrane was incubated at 4°C overnight with the primary monoantibody used at a dilution of 1 : 300 in TBS plus 0.1% Tween-20 (TBST). The membrane was washed several times with TBST and was subsequently incubated with HRP-conjugated secondary antibody (1 : 2000 in TBST) for 1 h at room temperature. After another wash of the membrane with TBST, the protein bands were detected using a supersensitive ECL solution (Boster Biological Technology Co. Ltd.) and visualized on an Amersham Imager 600 (GE Healthcare Life Sciences, Fairfield, USA).

### 4.7. DpdtbA Induced Changes in Lysosomal Membrane Permeability and Autophagy

The SGC-7901 cells were seeded into a 24-well flask and treated as described above for the cell viability assay. The cells were treated with different concentrations of DpdtbA (2.5 and 5.0 *μ*M DpdtbA) for 24 h. For detection of the acidic cellular compartment, acridine orange (or LysoTracker Red; Invitrogen) was used, which emits bright-red fluorescence in acidic vesicles but green fluorescence in the cytoplasm and nucleus. After treatment of the cells with the agent, acridine orange was then added at a final concentration of 1 *μ*g/ml (the concentration of LysoTracker Red, as recommended) for a period of 15 min. Following PBS washing, the fluorescent micrographs were captured using an inverted fluorescence microscope (Shanghai Lengguang Technology Co. Ltd., Shanghai, China) or a laser confocal fluorescence microscope (Zeiss LSM 510 Confocal Inverted Microscope, Jena, Germany).

### 4.8. Cell Cycle Analysis

The SGC-7901 or MGC-803 cells (1 × 10^5^) were seeded in a 6-well plate and incubated for 24 h at 37°C (5% CO_2_). The medium was replaced with fresh medium supplemented or not (control) with the agent (2.5 and 5.0 *μ*M). After 24 h of incubation, the cells were harvested with trypsin, followed by washing with PBS, fixed in 70% ethanol, and stored at −20°C. To stain the cellular nuclear DNA, the cells were suspended in 0.5 ml PBS containing 50 *μ*g/ml propidium iodide (PI) and 100 *μ*g/ml RNase after removing the 70% ethanol and washing with PBS. And then the cell suspension was incubated at 37°C for 30 min. DNA flow cytometry was performed in duplicate with a FACSCalibur flow cytometer (Becton Dickinson, USA). For each sample, 10,000 events were collected, and fluorescent signal intensity was recorded and analyzed by CellQuest and ModiFit (Becton Dickinson).

## Supplementary Materials

Supplementary Materials Figure S1: purity of DpdtbA was determined by HPLC. Figure S2: alteration of MMP with increased DpdtbA. (A) DMSO; (B) 2.5 *μ*M DpdtbA; (C) 5.0 *μ*M DpdtbA. Figure S3: the flow cytometric analysis of formation of autophagic vacuoles. (A) DMSO control; (B) 5 *μ*M DpdtbA; (C) 5.0 *μ*M DpdtbA + 1.5 mM 3-MA; (D) 5.0 *μ*M DpdtbA + 1.5 NAC. Figure S4: the microscopic analysis of formation of autophagic vacuoles. (A) DMSO control; (B) 5 *μ*M DpdtbA; (C) 5.0 *μ*M DpdtbA + 1.5 mM 3-MA; (D) 5.0 *μ*M DpdtbA + 1.5 NAC (objective size: 10 × 10).
