# Supplementary material for: Di-2-pyridylhydrazone Dithiocarbamate Butyric Acid Ester Exerted Its Proliferative Inhibition against Gastric Cell via ROS-Mediated Apoptosis and Autophagy
Source: Oxid Med Cell Longev. 2018 Mar 25;2018:4950705. doi: 10.1155/2018/4950705 (PMC5889906; doi:10.1155/2018/4950705)
Supplement: Supplementary Materials — Figure S1: purity of DpdtbA was determined by HPLC. Figure S2: alteration of MMP with increased DpdtbA. (A) DMSO; (B) 2.5 μM DpdtbA; (C) 5.0 μM DpdtbA. Figure S3: the flow cytometric analysis of formation of autophagic vacuoles. (A) DMSO control; (B) 5 μM DpdtbA; (C) 5.0 μM DpdtbA + 1.5 mM 3-MA; (D) 5.0 μM DpdtbA + 1.5 NAC. Figure S4: the microscopic analysis of formation of autophagic vacuoles. (A) DMSO control; (B) 5 μM DpdtbA; (C) 5.0 μM DpdtbA + 1.5 mM 3-MA; (D) 5.0 μM DpdtbA + 1.5 NAC (objective size: 10 × 10). [file 4950705.f1.zip › 4950705_SupplDesc.docx]

Supplementary materials

Purity of DpdtbA was determined by HPLC

Upon the structure of DpdtbA was characterized by NMR, the purity of it was further determined through HPLC (Shimadzu Corpation, Kyoto, Japan) technique (Fig. S1). The gradient condition as follows: 20-50% solvent B within 10 min, following increased to 100% in 10 min, and decreased to 20% in 2 min. finally keep same percent for 30 min. Solvent A: water plus 0.1% TFA; solvent B: acetonitrile plus 0.1% TFA.

Mitochondrial membrane permeability (MMP)

Mitochondrion is an important organelle that generates energy during oxidation of food, the integrity of mitochondrial membrane is crucial for cell life. When the membrane attacked by ROS, the cyt c release from mitochondrion was normally observed. To confirm the alteration of MMP, the rhodamine 123 (Sigma, USA), a popular green fluorescent mitochondrial dye was used for flow cytometry study. Following the company recommended protocol, the stained SGC-7901 cells that treated with or without DpdtbA (2.5 or 5.0 μM) were subjected to flow cytometric analysis (Becton-Dickinson, USA) (Fig. S2). As expected, more rhodamine 123 was retained in DpdtbA treated cells, indicating that the MMP was altered.

Flow cytometric and microscopic analyses of autophagic vacuoles

Cells were seeded into a 6-well plate and treated as described above for the cell viability assay. The cells were treated with either the agent alone (5.0 μM) or combination with (3-MA (1.5 mM) or NAC (1.5 mM)) for 24 h. Then the cell culture was removed, following PBS washing, trypsin digestion, finally the MDC (50 μM) were added as described previously [16]. The stained cells were subjected to flow cytometric analysis (Becton-Dickinson, USA) (Fig. S3). For morphologic analysis, autophagic vacuoles were labeled with MDC by incubating the cells that treated by either the agent or combination with 3-MA (or NAC) with 50 μM MDC in PBS at 37°C for 10 minutes. After incubation, cells were washed four times with PBS and immediately analyzed by fluorescence microscopy using an inverted microscope (Shanghai Lengguang Technology Co., Ltd., Shanghai, China) (Fig. S4).

Fig. S1 Purity of DpdtbA was determined by HPLC .

Fig. S2 Alteration of MMP with increased DpdtbA. (A) DMSO; (B) 2.5 µM DpdtbA; (C) 5.0 µM DpdtbA.

Fig.S3 The flow cytometric analysis of formation of autophagic vacuoles. (A) DMSO control; (B) 5 µM DpdtbA; (C) 5.0 µM DpdtbA + 1.5 mM 3-MA; (D) 5.0 µM DpdtbA + 1.5 NAC.

Fig.S4 The microcopic analysis of formation of autophagic vacuoles. (A) DMSO control; (B) 5 µM DpdtbA; (C) 5.0 µM DpdtbA + 1.5 mM 3-MA; (D) 5.0 µM DpdtbA + 1.5 NAC. (objective size: 10 ×10).
