# Supplementary figures and images for: Di-2-pyridylhydrazone Dithiocarbamate Butyric Acid Ester Exerted Its Proliferative Inhibition against Gastric Cell via ROS-Mediated Apoptosis and Autophagy
Source: Oxid Med Cell Longev. 2018 Mar 25;2018:4950705. doi: 10.1155/2018/4950705 (PMC5889906; doi:10.1155/2018/4950705)

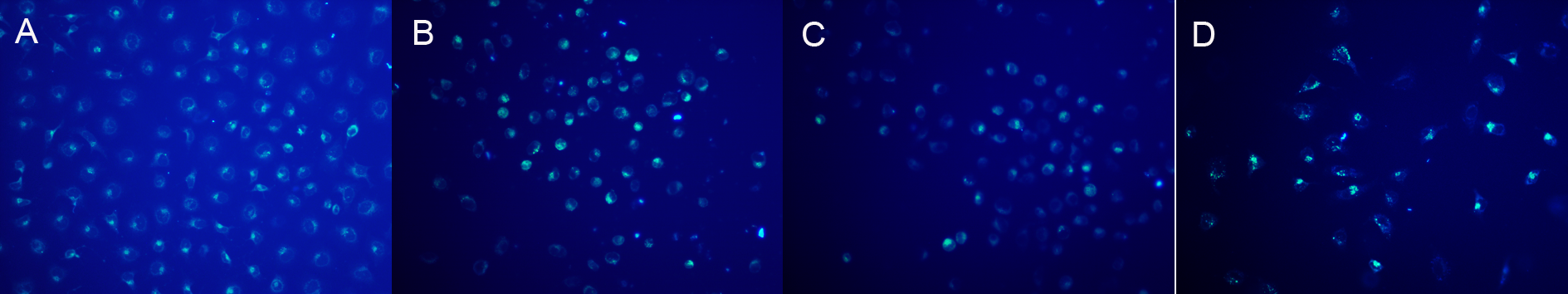

Supplement: Supplementary Materials — Figure S1: purity of DpdtbA was determined by HPLC. Figure S2: alteration of MMP with increased DpdtbA. (A) DMSO; (B) 2.5 μM DpdtbA; (C) 5.0 μM DpdtbA. Figure S3: the flow cytometric analysis of formation of autophagic vacuoles. (A) DMSO control; (B) 5 μM DpdtbA; (C) 5.0 μM DpdtbA + 1.5 mM 3-MA; (D) 5.0 μM DpdtbA + 1.5 NAC. Figure S4: the microscopic analysis of formation of autophagic vacuoles. (A) DMSO control; (B) 5 μM DpdtbA; (C) 5.0 μM DpdtbA + 1.5 mM 3-MA; (D) 5.0 μM DpdtbA + 1.5 NAC (objective size: 10 × 10). [file 4950705.f1.zip › Fig.S4_OMCL_2190463.tif]

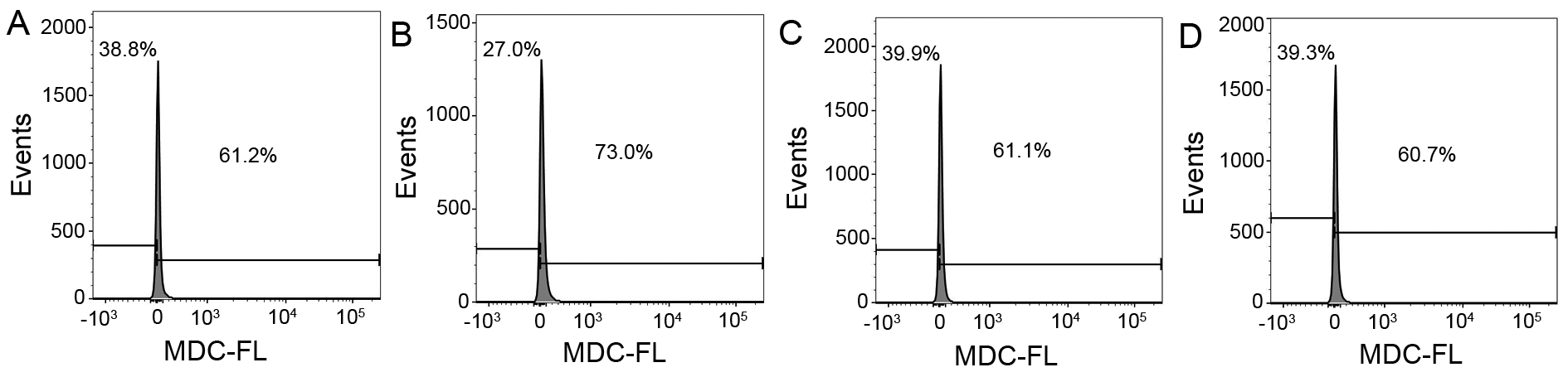

Supplement: Supplementary Materials — Figure S1: purity of DpdtbA was determined by HPLC. Figure S2: alteration of MMP with increased DpdtbA. (A) DMSO; (B) 2.5 μM DpdtbA; (C) 5.0 μM DpdtbA. Figure S3: the flow cytometric analysis of formation of autophagic vacuoles. (A) DMSO control; (B) 5 μM DpdtbA; (C) 5.0 μM DpdtbA + 1.5 mM 3-MA; (D) 5.0 μM DpdtbA + 1.5 NAC. Figure S4: the microscopic analysis of formation of autophagic vacuoles. (A) DMSO control; (B) 5 μM DpdtbA; (C) 5.0 μM DpdtbA + 1.5 mM 3-MA; (D) 5.0 μM DpdtbA + 1.5 NAC (objective size: 10 × 10). [file 4950705.f1.zip › Fig.S3_OMCL_2190462.tif]

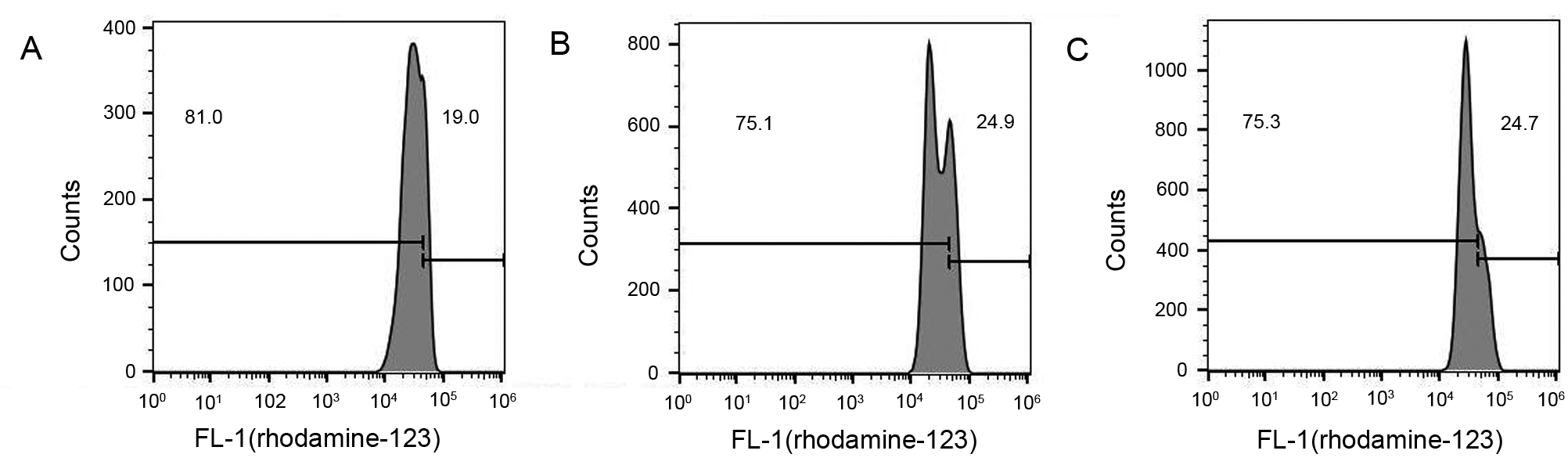

Supplement: Supplementary Materials — Figure S1: purity of DpdtbA was determined by HPLC. Figure S2: alteration of MMP with increased DpdtbA. (A) DMSO; (B) 2.5 μM DpdtbA; (C) 5.0 μM DpdtbA. Figure S3: the flow cytometric analysis of formation of autophagic vacuoles. (A) DMSO control; (B) 5 μM DpdtbA; (C) 5.0 μM DpdtbA + 1.5 mM 3-MA; (D) 5.0 μM DpdtbA + 1.5 NAC. Figure S4: the microscopic analysis of formation of autophagic vacuoles. (A) DMSO control; (B) 5 μM DpdtbA; (C) 5.0 μM DpdtbA + 1.5 mM 3-MA; (D) 5.0 μM DpdtbA + 1.5 NAC (objective size: 10 × 10). [file 4950705.f1.zip › Fig.S2_OMCL_2190461.tif]

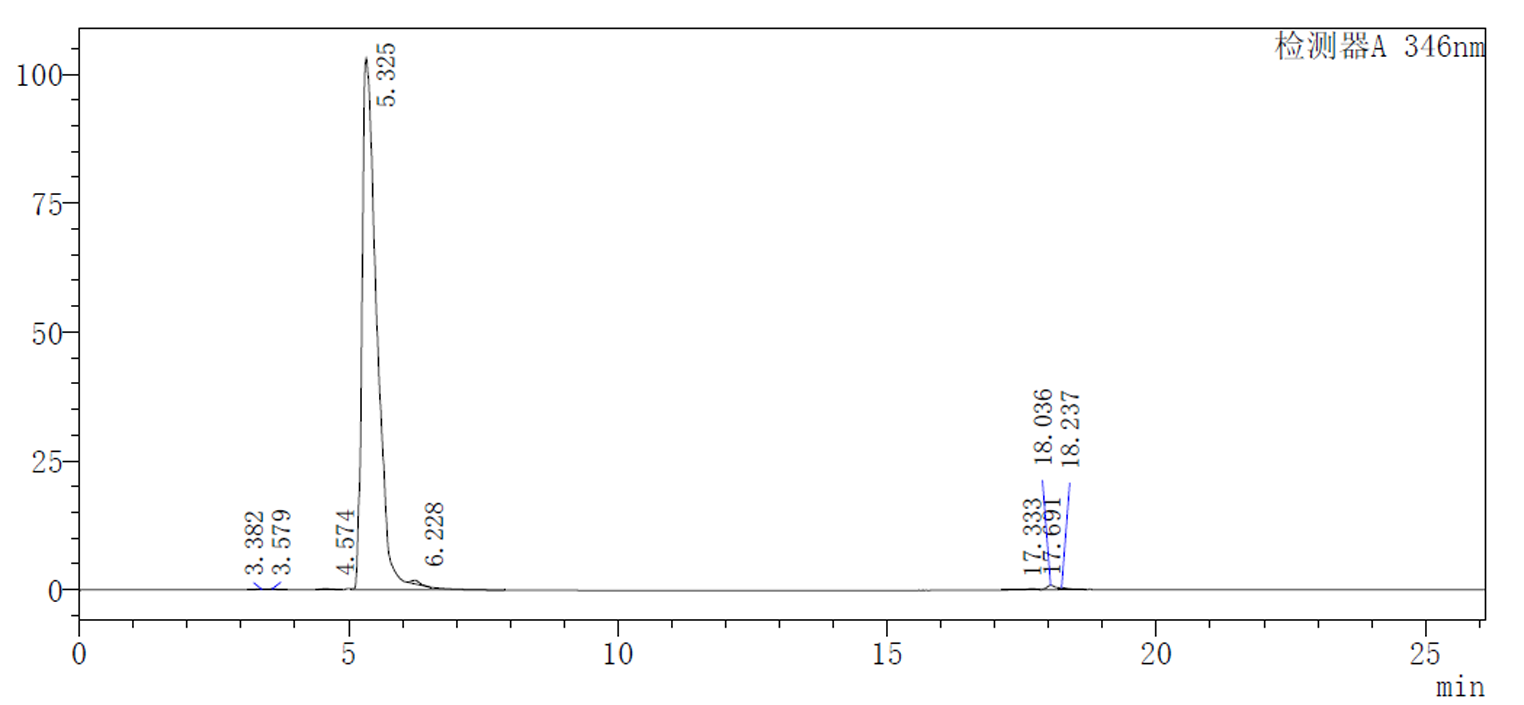

Supplement: Supplementary Materials — Figure S1: purity of DpdtbA was determined by HPLC. Figure S2: alteration of MMP with increased DpdtbA. (A) DMSO; (B) 2.5 μM DpdtbA; (C) 5.0 μM DpdtbA. Figure S3: the flow cytometric analysis of formation of autophagic vacuoles. (A) DMSO control; (B) 5 μM DpdtbA; (C) 5.0 μM DpdtbA + 1.5 mM 3-MA; (D) 5.0 μM DpdtbA + 1.5 NAC. Figure S4: the microscopic analysis of formation of autophagic vacuoles. (A) DMSO control; (B) 5 μM DpdtbA; (C) 5.0 μM DpdtbA + 1.5 mM 3-MA; (D) 5.0 μM DpdtbA + 1.5 NAC (objective size: 10 × 10). [file 4950705.f1.zip › Fig.S1-HPLC_OMCL_2190460.tif]
